# Niche Overlap Between Two Sympatric Steppe Birds in Inner Mongolia: Habitat Selection and Insights for Conservation

**DOI:** 10.1002/ece3.71010

**Published:** 2025-02-24

**Authors:** Zheng Han, Xi Yang, Lishi Zhang, Frédéric Jiguet, Piotr Tryjanowski, Haitao Wang

**Affiliations:** ^1^ School of Life Sciences Northeast Normal University Changchun China; ^2^ College of Agricultural Hulunbuir University Hulunbuir China; ^3^ Animal's Scientific and Technological Institute Agricultural University of Jilin Changchun China; ^4^ CESCO UMR7204 MNHN‐CNRS‐Sorbonne Université Paris France; ^5^ Department of Zoology Poznań University of Life Sciences Poznań Poland; ^6^ Jilin Provincial Key Laboratory of Animal Resource Conservation and Utilization Northeast Normal University Changchun China; ^7^ Jilin Engineering Laboratory for Avian Ecology and Conservation Genetics Northeast Normal University Changchun China

**Keywords:** endangered, habitat selection, Jankowski's bunting, meadow bunting, niche overlap and segregation, steppe birds

## Abstract

The destruction and degradation of natural ecosystems is a major driver of biodiversity loss. The steppe ecosystem is under threat from human activities and habitat degradation. Fine‐scale breeding habitat selection is critical for the survival of steppe birds, but understanding the factors that drive this selection remains challenging. This study uses field point‐count surveys to examine factors influencing habitat selection and quantify niche overlap between two closely related steppe bird species: Jankowski's Bunting ( *Emberiza jankowskii* ) and Meadow Bunting ( *E. cioides* ) in Inner Mongolia, China. These species share similar ecological traits and overlapping habitats, making them ideal for exploring how fine‐scale habitat selection and resource differentiation enable coexistence despite ecological similarity. We use generalized linear models (GLMs) and niche modeling algorithms to analyze the data. The results reveal distinct habitat preferences at both local and landscape scales. Jankowski's Bunting favors areas with higher vegetation cover and height, while Meadow Bunting prefers sites with greater edge density. GLM results show non‐linear responses of both species to habitat variables, with distinct thresholds for optimal occurrence. Niche overlap analysis indicates considerable overlap (Schoener's D = 0.57), but significant differences in niche centroids suggest niche differentiation between the two species. Our findings emphasize the importance of considering fine‐scale habitat characteristics and non‐linear species –habitat relationships in conservation planning for steppe birds. Understanding how these species respond to habitat changes resulting from anthropogenic activities—such as land‐use conversion and agricultural intensification—can help tailor conservation efforts to mitigate negative impacts and promote species coexistence in sensitive habitats.

## Graphical abstract

Niche overlap patterns for Jankowski's Bunting and Meadow Bunting. These species share similar ecological traits and overlapping habitats, making them ideal for exploring how fine‐scale habitat selection and resource differentiation enables coexistence despite ecological similarity.

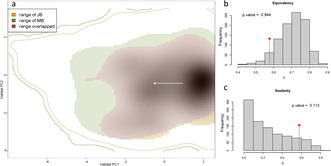

## 1 Introduction

Destruction and degradation of natural ecosystems are the primary drivers of biodiversity loss (Rands et al. 2010; Pereira et al. 2012). Steppe ecosystems, characterized by expansive grasslands interspersed with shrubs and sparse trees, provide critical habitats for many bird species adapted to these environments (Donald et al. 2006; Butler et al. 2010). Agricultural intensification, climate change, and human disturbance have contributed to the decline of steppe bird populations (Stanton et al. 2018; Rosenberg et al. 2019). As these habitats become fragmented and converted for human use, grassland birds lose nesting sites and foraging grounds. Habitat selection involves behavioral decisions made by individuals regarding environmental features at various spatial scales to maximize fitness and survival (Orians and Wittenberger 1991). Highly mobile organisms, such as birds, first assess habitat suitability at large landscape scales. At this scale, birds typically select a general area based on factors such as regional climate or habitat fragmentation. They then refine their habitat choices at finer scales, selecting specific home ranges and nest sites (McGarigal et al. 2016; Amini Tehrani et al. 2020). Birds may exhibit varying associations with habitat features, social cues, or resources across different spatial scales. Relying on observations from a single spatial scale to draw conclusions about habitat selection can overlook critical factors operating at other scales (Cornell and Donovan 2010).

Habitat structure, particularly vegetation, is a key factor influencing birds' foraging, reproduction, and survival, both directly and indirectly (Rotenberry and Wiens 1980). Species generally exhibit preferences for specific vegetation types or heights. For example, Yellowhammers ( *Emberiza citrinella* ) preferred areas with denser vegetation and closer to productive farmland feeding areas than sympatric Ortolan Buntings ( *E. hortulana* ) in Norway (Dale and Manceau 2003). Species like Lark Sparrows ( *Chondestes grammacus* ) and Horned Larks ( *Eremophila alpestris* ) depend on early successional habitats, typically characterized by short vegetation and limited woody encroachment. In contrast, co‐occurring species such as Clay‐colored Sparrows ( *Spizella pallida* ) and Henslow's Sparrows ( *Ammodramus henslowii* ) prefer grasslands in later successional stages, often with a shrub component (Grant et al. 2004; Winter et al. 2005). Additionally, the response of grassland birds to local features generally depends on the landscape context. For example, the species richness of grassland specialists is positively correlated with the proportion of grassland in the surrounding landscape (Canonne et al. 2024). Birds tend to avoid habitat edges in fragmented grassland habitats (Besnard et al. 2016). These findings highlight the importance of considering both local habitat characteristics and broader landscape features to understand species habitat selection processes. Studies also indicate that the importance of spatial scales varies among species and functional groups (Coreau and Martin 2007; Vergara and Armesto 2009; Banks‐Leite et al. 2013). Habitat generalists are generally less influenced by landscape structure compared to habitat specialists (Devictor et al. 2008). Species that rely on complementary resources from different locations are more influenced by landscape configuration, whereas those dependent on small, uniform patches (e.g., individual ponds) are more affected by local‐scale factors (Fahrig et al. 2011; Pérez‐García et al. 2014). These findings suggest that the importance of different scales in habitat selection is related to species' life‐history traits and ecological requirements.

Hutchinson's n‐dimensional hypervolume concept defines the ecological niche as the range of conditions that allow a species to survive and reproduce (Hutchinson 1957). This concept offers a simple geometric interpretation of the niche. Recent advances in niche modeling enable efficient quantification and comparison of species' niches by calculating the intersection of their hyper‐volumes using distance metrics and similarity indices (Mammola 2019). Sympatric species often compete for essential resources such as food, habitat, and nutrients (Schoener 1983). In response to competition, species may divide their niches by differing in their use of microhabitats, resources, or timing (Surya and Keitt 2019; Kent and Sherry 2020; Dehling et al. 2021). Research on closely related bird species has shown that niche differentiation often leads to habitat segregation, either between distinct habitats or within different areas of the same habitat (Jones 2001; Surya and Keitt 2019). Jankowski's Bunting and Meadow Bunting are two small passerine bird species mainly found in Asia. Jankowski's Bunting is a rare species endemic to the steppes of Inner Mongolia and neighboring regions (Wang et al. 2011). This elusive species exhibits a specific nesting ecology, preferring open grassland habitats with scattered shrubs and tall grasses (Zhang et al. 2022). In contrast, Meadow Bunting has a wider distribution across East Asia, including China, Japan, Korea, and parts of Russia (BirdLife International 2024a). It prefers breeding in open grasslands, meadows, agricultural fields, and forest edges, often nesting in shrubs or low vegetation (Deng et al. 2003). The breeding population of Jankowski's Bunting is currently estimated at between 9800 and 12,500 individuals, with suitable breeding habitat covering approximately 280 km^2^ (Han et al. 2018). This species is classified as “Endangered” by the International Union for Conservation of Nature (IUCN) due to rapid population decline and continued contraction of its breeding range (BirdLife International 2024b). In contrast, the population trend of Meadow Bunting appears stable or varies slightly across regions (Choi et al. 2020). This species does not meet the criteria for “Vulnerable” based on range size or population trend and is therefore classified as “Least Concern” by the IUCN (BirdLife International 2024a).

Jankowski's Bunting and Meadow Bunting are closely related species with overlapping ranges, but they likely have distinct breeding habitat preferences and occupy similar ecological niches. Understanding the mechanisms that governing their habitat selection, as well as niche overlap or segregation, are crucial to understand the dynamics of their coexistence and population trends. We conducted point count surveys in steppe habitats to document the characteristics of breeding sites selected by both species. Using generalized linear models (GLMs) and niche modeling algorithms, the study aimed to: (1) identify key factors influencing habitat selection in Jankowski's Bunting and Meadow Bunting, and (2) quantify the degree of niche overlap or segregation between the two species. This may help predict how these buntings and other coexisting bird species could be affected by future agricultural changes.

## 2 Materials and Methods

### 2.1 Study Area and Species Occurrence Data

**FIGURE 1:**
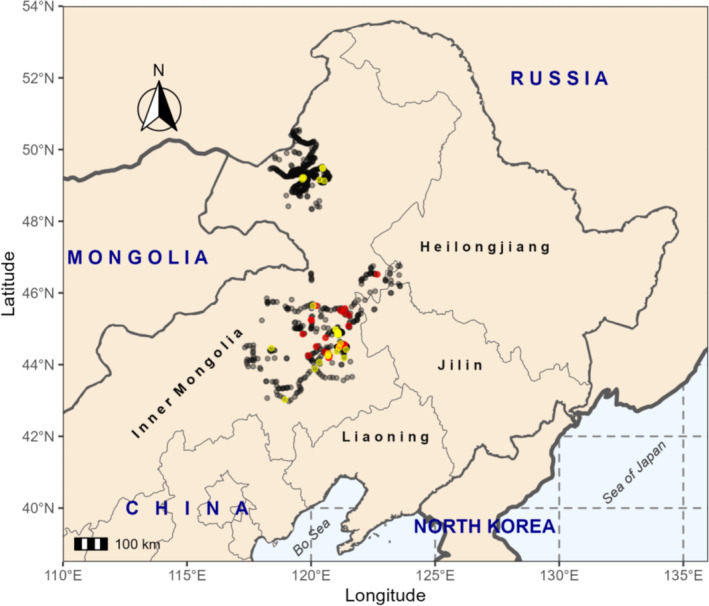
The presence sites of Jankowski's Bunting (in red) and Meadow Bunting (in yellow), gray dots indicate the absence sites that are located within a 100‐km distance from any recorded presence points.

The research was conducted in the eastern region of Inner Mongolia, China (Figure 1), characterized by a continental, temperate climate with an average annual temperature of 6°C–7°C and annual rainfall of 300–400 mm, mainly occurring from April to September, coinciding with the vegetation period (Wu et al. 2015). The study area includes three primary vegetation zones: temperate coniferous and deciduous forests, meadow steppe, and typical steppe (Han et al. 2009). Additionally, the landscape contains scattered fragments of croplands, bare lands, tree plantations, and human settlements.

From 2018 to 2022, we surveyed 1500 distinct sampling sites within the study area. These points were randomly distributed across different land cover types but were subject to specific spatial constraints: they were at least 100 m away from clear‐cuts and large obstacles (such as urban or suburban developments and roads) and were separated by a minimum distance of 500 m. This ensures that the sampling points were distributed in a manner that represented the variability within the landscape while minimizing potential biases from nearby human‐made disturbances. This spatial separation also reduces the risk of double‐counting of birds, ensuring that each point represents a unique sampling location (Han et al. 2021).

From April 20 to July 10, the peak breeding season for steppe birds in Inner Mongolia, we assessed the distribution of Jankowski's Bunting and Meadow Bunting using the standard point‐count method (Bibby et al. 2000). Trained observers remained stationary at each point for 10 min and recorded all bird species observed or heard within a 100‐m radius. Birds in flight were counted only if actively engaged within the survey area (e.g., foraging or displaying) (Han et al. 2021). Point counts were conducted within 4 h of sunrise, avoiding adverse weather conditions (e.g., heavy rain or strong winds). Each site was surveyed twice in one single year: once before and once after mid‐May, with a 4–6 week interval between surveys. Species presence was recorded if detected during at least one survey. It should be noted that not all 1500 sites were sampled every year. Instead, sampling efforts were distributed almost evenly across the 5 years, with an average of approximately 300 sites sampled annually.

### 2.2 Habitat Variables

Habitat data were collected once per year, after conducting the second bird survey at each site. For the local‐scale habitat variables, three 2 − 2 m quadrats were placed within a 10 m radius at each sampling site (Han et al. 2020). The habitat types within these three quadrats are generally homogeneous because the sampling sites were selected away from any abrupt habitat edges. We recorded three habitat variables within each quadrat: plant canopy, plant richness, and plant height. Plant canopy was visually estimated from a distance of 4 m and at a height of 1 m in front of a Robel pole. Plant richness was quantified as the number of distinct plant species present in a quadrat. Plant height was measured as the average of plant heights taken at 10 random points along a diagonal line within the quadrat. The values from the three quadrats were averaged for each sampling point to yield a representative measure of the local habitat. These variables offer insights into vegetation structure, which is crucial for nesting and foraging by steppe birds (Han et al. 2021).

We obtained a Landsat‐derived annual China Land Cover Dataset (CLCD) with a 30‐m resolution for 2019 to assess landscape‐scale habitat variables. The CLCD categorized land cover into nine distinct types: cropland, forest, shrub, grassland, water, snow and ice, barren, impervious surfaces, and wetland, achieving an overall accuracy of 79.31% (Yang and Huang 2021). We calculated six variables to describe habitat composition and configuration: (a) edge density; (b) patch density; (c) largest patch index; (d) total core area index; (e) grassland proportion (%); and (f) Simpson diversity of land cover types. Detailed explanations of these variables can be found in Table S2. These indices were calculated within a 300‐m radius from each sampling point using the R package “landscapemetric” (Hesselbarth et al. 2019). The 300‐m radius was selected to minimize overlap between adjacent buffered areas and reduce redundancy in landscape attributes at closely spaced points. Furthermore, this radius approximates the typical territory size of many songbirds, offering a meaningful scale for landscape habitat analysis.

In addition to the CLCD data, we acquired the annual maximum Normalized Difference Vegetation Index (NDVI) for each sampling point, a widely used remote sensing metric that quantifies vegetative density (Cuomo et al. 2001). The NDVI dataset, which has a 30‐m resolution and spans from 2000 to 2020, was accessed from the National Earth System Data Center (https://doi.org/10.12199/nesdc.ecodb.rs.2021.012). To ensure consistency with the CLCD data, we utilized the 2019 NDVI values for analysis. We assumed that vegetation structure and NDVI at each site remained relatively stable during each breeding season, and annual trends were not analyzed due to the short duration of our study.

### 2.3 Breeding Habitat Selection

We investigated the breeding habitat selection of Jankowski's and Meadow Buntings by comparing environmental variables measured at presence and pseudo‐absence points. Selecting appropriate pseudo‐absence or background locations is a critical step in species distribution modeling (SDM) (Chefaoui and Lobo 2008). One common approach involves selecting locations where other species are present, but the target species is absent (e.g., Elith and Leathwick 2007). A previous study examined the relationship between the geographic extent of pseudo‐absence selection and model performance using 12 species from the Australian Wet Tropics. The study found that model performance decreased when pseudo‐absence points were drawn from either very restricted or overly broad regions compared to the species' actual occurrence data. Specifically, the AUC increased rapidly as the background size expanded from 10 to 100 km, with only marginal increases beyond this range. For example, by 100 km, all models had an AUC greater than 0.93, and at 500 km, the AUC exceeded 0.99 (VanDerWal et al. 2009). In our study, we selected a subset of absence points located within 100 km of recorded presence points, designating them as “pseudo‐absence” sites. We aimed to focus on areas where the absence of the species is more likely related to local environmental conditions rather than solely to dispersal constraints (Senay et al. 2013). If pseudo‐absences are located too far from known occurrences, the resulting models may be dominated by broad regional factors. This limitation can hinder the model's ability to capture fine‐scale environmental conditions that directly influence species distribution. This 100‐km threshold can also reduce over‐prediction and extrapolation when assessing bird habitat suitability (Brotons et al. 2004).

We conducted an ANOVA (Analysis of Variance) test to determine whether the mean values of the 10 habitat variables inhabited by Jankowski's Bunting and Meadow Bunting differ. ANOVA assesses variability within and between groups, helping researchers determine whether observed differences are due to chance or indicate true effects.

Given the high correlations among the 10 habitat variables (Figure S1), we initially conducted Principal Components Analysis (PCA) using the R package “ade4” to reduce dimensionality (Thioulouse et al. 2018). The first two principal components (PCs) accounted for 62.1% of the total variation in the habitat data and were retained for subsequent analysis (Table S3). To investigate the relationship between species occurrence and habitat variation, we developed two Generalized Linear Models (GLMs) with binomial distributions. The occurrence of Jankowski's Bunting and Meadow Bunting served as response variables, while the habitat PCs acted as explanatory variables (Zuur et al. 2007). We also incorporated quadratic terms for the habitat PCs to capture potential nonlinear relationships, which are common in ecological systems where species responses to environmental gradients may show curvature (Suárez‐Seoane et al. 2002; Jamil and Ter Braak 2013; Anderson et al. 2022). This approach enables a better understanding of whether bird occurrence peaks or declines at specific levels of habitat variation, offering additional insights into their ecological preferences. To visualize these relationships, we generated response surfaces and inflated response curves using the R package “lattice” (Sarkar et al. 2015). Response surfaces illustrate both the direction and magnitude of the relationships while capturing any curvature. Inflated response curves, an extension of partial dependence plots, demonstrate the effect of each PC on species occurrence, considering both the average and extreme values of the other variables (Zurell et al. 2012). This method enhances model accuracy and interpretability by identifying potential thresholds or optimal conditions for species occurrence within the habitat gradient.

### 2.4 Niche Comparison

We followed the methodology proposed by Broennimann et al. (2012) to quantify and compare the habitat niches of Jankowski's Bunting and Meadow Bunting, two sympatrically breeding and closely related species. As previously described, we first used Principal Components Analysis (PCA) to condense the environmental space defined by 10 habitat variables into a simplified two‐dimensional representation. This involved calibrating the PCA model with habitat data from various sites, which served as background data (Senay et al. 2013). Subsequently, we projected the occurrences of each species onto a grid of cells bounded by the minimum and maximum PCA scores in the study areas (Broennimann et al. 2012; Ralston et al. 2017; Lu and Jetz 2023). We then estimated the smoothed density of species occurrences within each grid cell, using the kernel density function to correct for potential sampling bias in occurrence records.

To quantify niche overlap, we used Schoener's D metric (Schoener 1970), which provides a comprehensive measure of niche correspondence across the entire environmental spectrum, ranging from complete non‐overlap (0) to complete overlap (1). Our assessment of niche equivalency and similarity followed the randomized testing framework outlined by the “ecospat” package, which evaluates the statistical significance of measured niche differences against null model niches randomly selected from a specified background area (Di Cola et al. 2017). These functions have been widely used to compare niches among sister species (Silva et al. 2014; Villegas et al. 2021). We ran the model with default settings and repeated each randomization process 1000 times, producing a null distribution of overlap values against which the observed score was compared. This method can assess whether the niches of these two species across different geographical ranges are equivalent, meaning whether the niche overlap remains consistent when randomly reallocating the occurrences of both species among the ranges. Niches are considered more equivalent than expected by chance if the *p*‐value exceeds 0.95 (Graham et al. 2004). Conversely, a niche similarity test evaluates whether the environmental niche occupied in one range is more similar to that in another range than expected by chance. Niches are considered more similar than expected by chance if the *p*‐value is below 0.05.

## 3 Results

From 2018 to 2022, we conducted bird surveys at 1500 distinct sampling sites within the study area. Among these, Jankowski's Bunting exhibited breeding behavior in 70 sites, and Meadow Bunting in 58 sites, with one site shared by both species. Subsequently, we identified 1083 absence sites within a 100‐km radius of any documented presence points (Figure 1).

**FIGURE 2:**
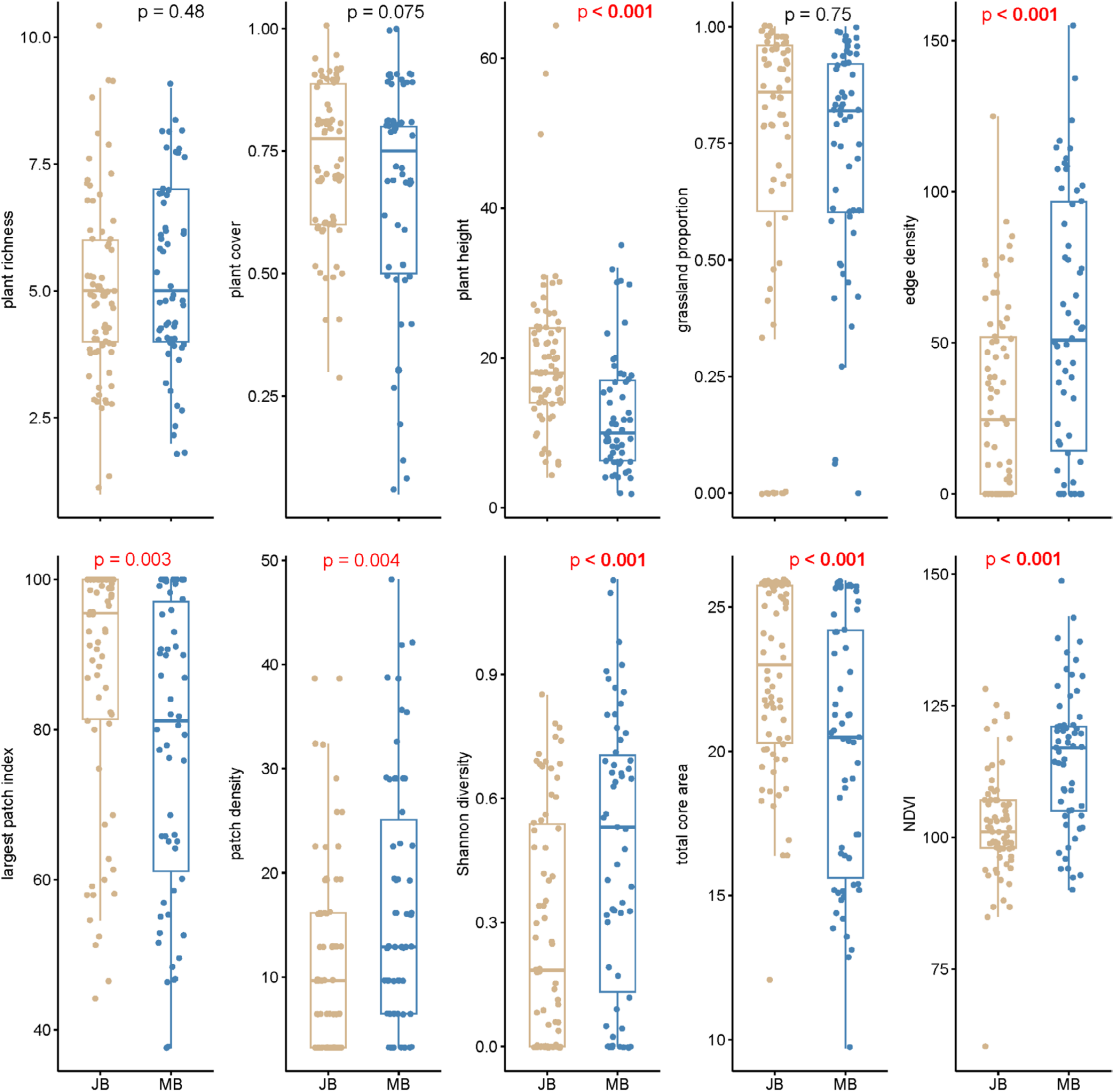
Boxplots to show the differences between the habitat variables of sites inhabited by Jankowski's Bunting (JB) and Meadow Bunting (MB). The *p*‐value displayed in red means a statistically significant difference existing between the two species according to the ANOVA tests.

Analysis of habitat variables revealed significant disparities between sites inhabited by Jankowski's Bunting and Meadow Bunting at both local and landscape scales (Figure 2 and Table S1). Locally, Jankowski's Bunting occupied areas with higher mean values of plant cover and plant height than those of Meadow Bunting. On a broader scale, sites inhabited by Jankowski's Bunting exhibited a greater largest patch index and total core area. ANOVA tests revealed significant differences in most landscape‐scale variables, except for grassland proportion; however, significant differences were only observed in plant height at the local scale (Figure 2; Table S1).

**FIGURE 3:**
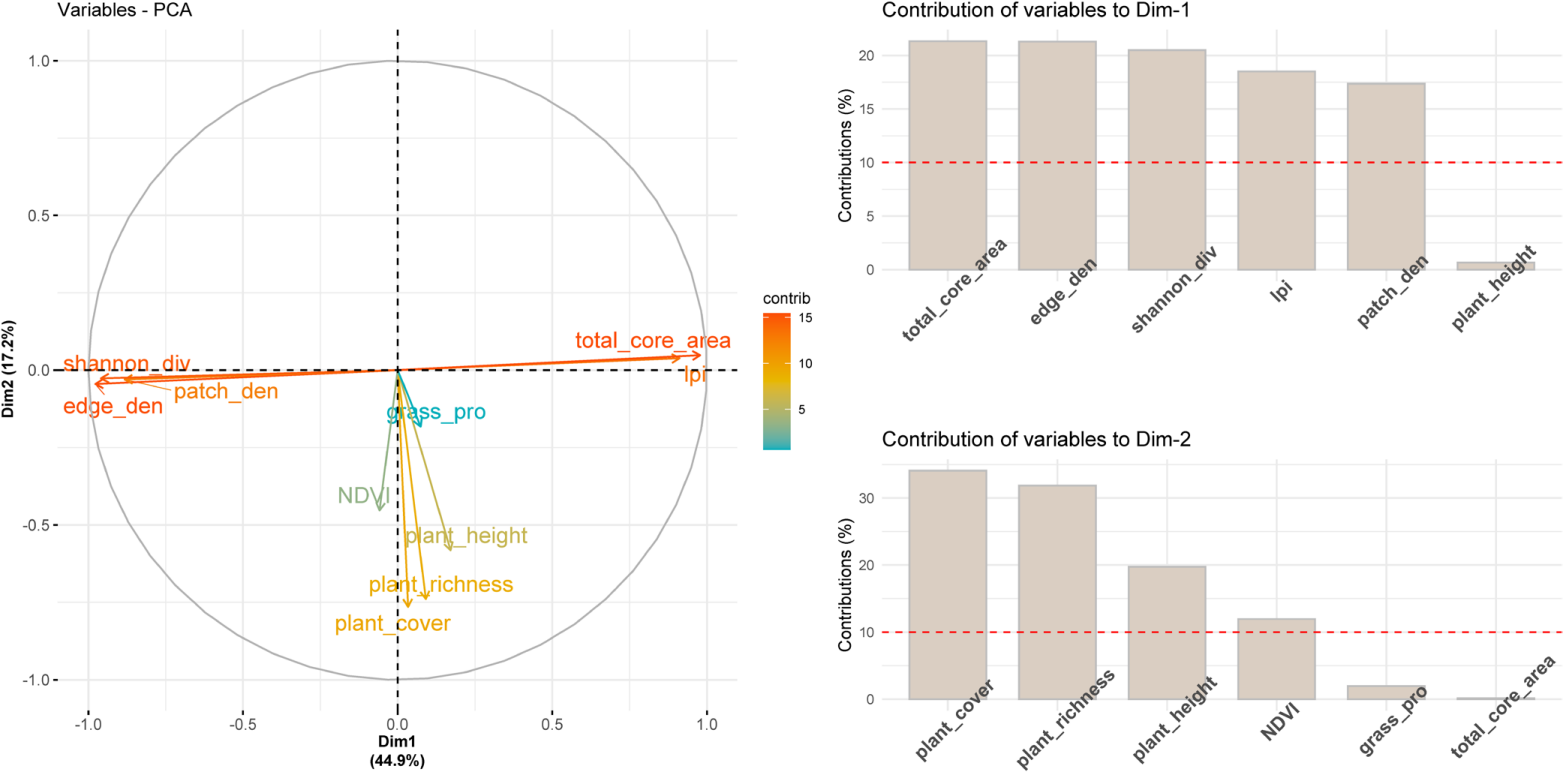
Principal component analysis (PCA) results of habitat variables assessed at each sampling site. Left panel: Variable correlation plots. Positive correlated variables point to the same side of the plot. Negative correlated variables point to opposite sides. Right panel: Barplots showing the contributions of variables to the first two principal components (Dim‐1 and Dim‐2) are expressed in percentage. The red dashed line on the graph indicates the expected average contribution. For a given component, a variable with a contribution larger than this cutoff could be considered as important in contributing to the component. For simplicity, only the top six contributing variables are shown here.

Principal Component Analysis (PCA) revealed that the first two components (habitatPC1 and habitatPC2) accounted for 44.9% and 17.2% of the total variability, respectively (Figure 3; Table S3). HabitatPC1 showed positive correlations with total core area and largest patch index but negative associations with edge density, patch density, and Shannon diversity of land cover types, indicating a gradient of habitat integrity at the landscape scale. Conversely, habitatPC2 represented an inverse gradient of habitat quality, characterized by negative correlations with plant cover, plant richness, plant height, grassland proportion, and NDVI (Figure 3; Table S3).

**FIGURE 4:**
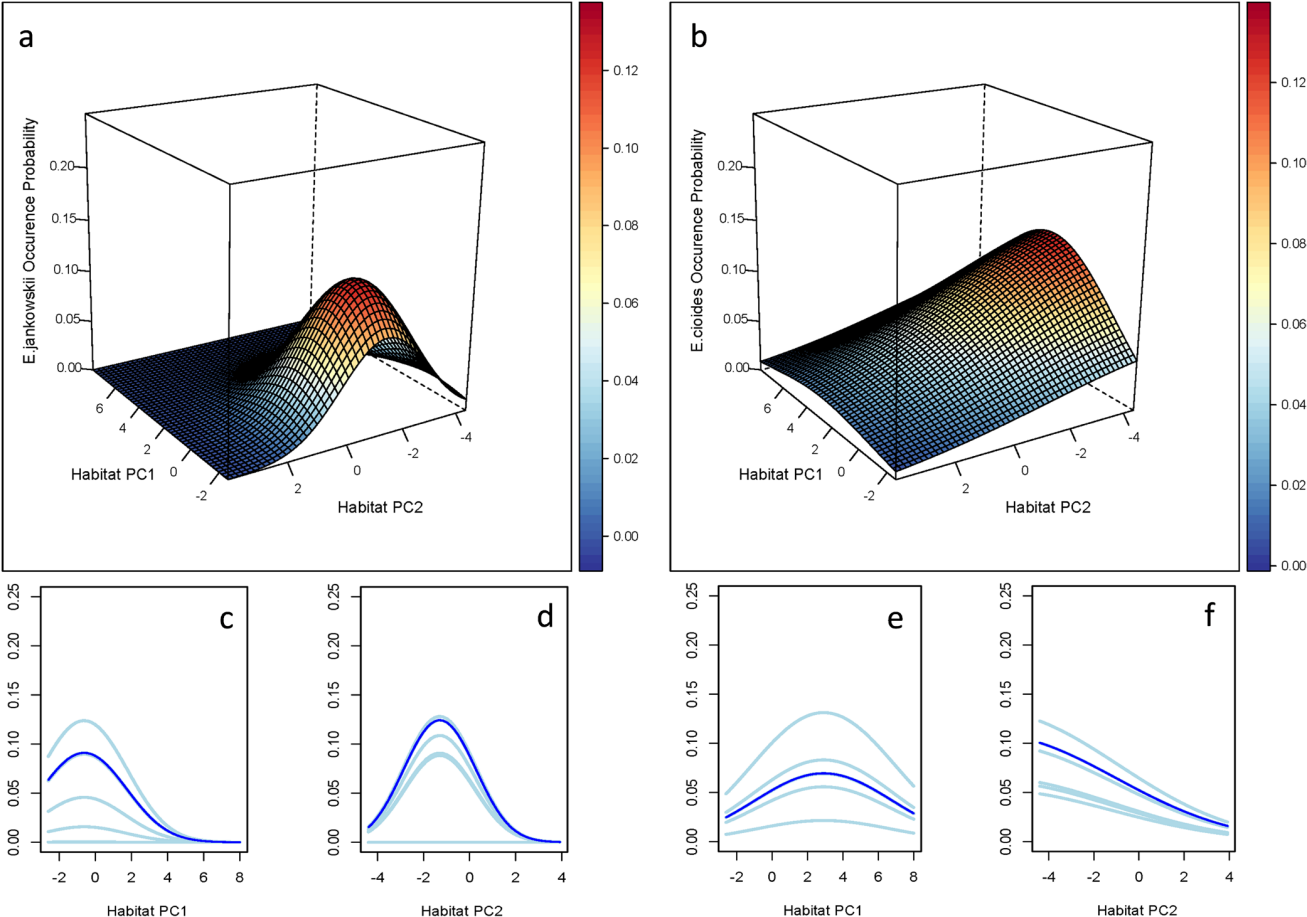
Response surfaces (a, b) and inflated response curves (c–f) show how the occurrence probability of Jankowski's Bunting (left) and Meadow Bunting (right) varies with habitat variables (Habitat PC1 and Habitat PC2). Dark lines are the mean response curves, and light lines depict the habitat effects over the full range of the other predictors (minimum, maximum, median and quartiles).

In the fitted Generalized Linear Models (GLM), the coefficients associated with quadratic terms for both Jankowski's Bunting and Meadow Bunting are non‐zero, indicating that their occurrences respond non‐linearly to changes in habitat variables (Table 1). The inflated response curves indicate that the occurrence probability of Jankowski's Bunting responds optimally to intermediate levels of habitat PC1 and PC2, rather than exhibiting a linear or monotonic relationship. Conversely, the occurrence rate of Meadow Bunting peaked along habitat PC1 but showed a linear decrease with increasing habitat PC2 (Figure 4). Although the species displayed similar unimodal response patterns, specific thresholds or optimal conditions were evident for each species. For instance, Jankowski's Bunting reached its peak occurrence at a habitat PC1 value of 0, while Meadow Bunting peaked at a value of 3 (Figure 4). The response surfaces further confirmed the non‐linear species –habitat relationship, with both species displaying concave‐downward response patterns. This indicates that changes in the habitat gradient lead to progressively smaller changes in species occurrence probability (Figure 4).

**TABLE 1:** Results of the binomial GLM analysis: Relationships between the occurrence probability of two closely related steppe birds (Jankowski's Bunting and Meadow Bunting) and derived habitat principal components in north‐eastern Inner Mongolia.

	Jankowski's bunting				Meadow bunting			
	Est	95% CI	*Z* value	Pr(>|z|)	Est	95% CI	*Z* value	Pr(>|z|)
(Intercept)	−2.339	(−2.763, −1.935)	−11.080	< 0.001***	−2.897	(−3.297, −2.517)	−14.565	< 0.001***
HabitatPC1	−0.124	(−0.285, 0.027)	−1.576	0.115	0.208	(0.049, 0.376)	2.502	0.012*
I(habitatPC1^2^)	−0.100	(−0.202, −0.015)	−2.090	0.037*	−0.036	(−0.094, 0.013)	−1.318	0.187
HabitatPC2	−0.599	(−0.930, −0.318)	−3.860	< 0.001***	−0.241	(−0.480, −0.029)	−2.108	0.035*
I(habitatPC2^2^)	−0.232	(−0.416, −0.081)	−2.718	0.007**	−0.018	(−0.146, 0.089)	−0.306	0.759
AIC		510.4				463.3		
*F*		5.219				2.895		
RMSE		0.23				0.21		

Abbreviations: 95% CI, 95% confidence intervals; Est, Estimation. ***p < 0.001, **p < 0.01, *p < 0.05.

**FIGURE 5:**
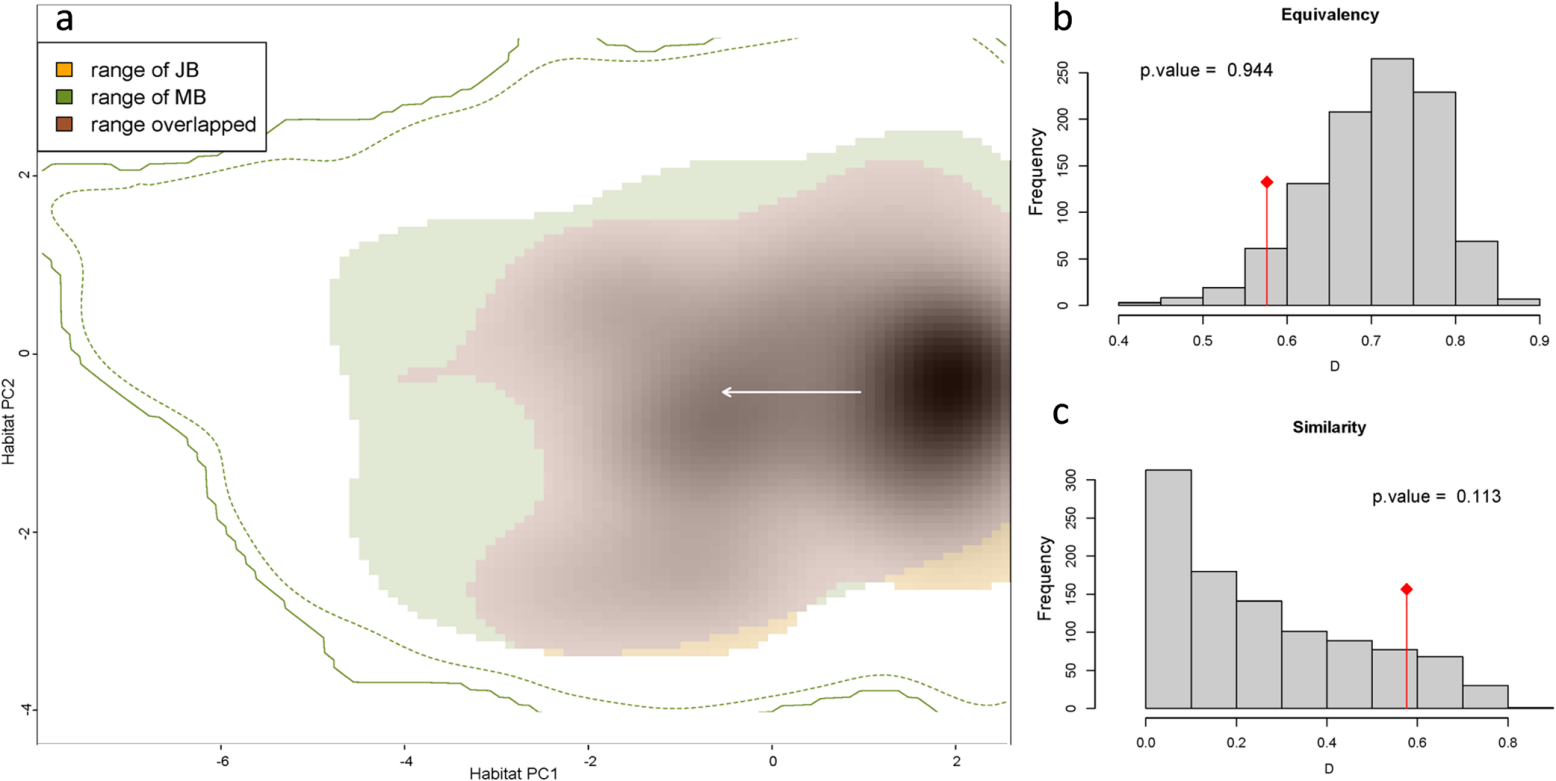
Left panel: Density plot a represents the niche overlap patterns for Jankowski's Bunting and Meadow Bunting, along the two‐first axes of habitat PCA. The solid and dashed contour lines represent 100% and 90% of the available (background) environment, respectively. The densities of occurrences of Jankowski's Bunting by cell are displayed in the gray shade. The white arrow links the distribution centroid of Jankowski's Bunting (on the right side) and Meadow Bunting (on the left side). Right panel: Histograms show the observed niche overlap D (bars with a diamond) and simulated niche overlaps (gray bars), based on tests of niche equivalency (b), and niche similarity (c). The significances (*p*‐value) of the two tests are also shown.

The endangered Jankowski's Bunting occupies a narrower niche range than that of Meadow Bunting (Figure 5; Figure S2). There is considerable overlap in the environmental space occupied by both species (Schoener's D = 0.57), despite significant differences in their niche centroids (Figure 5). Our analysis reveals that the two related species rarely have identical niches (*p* = 0.944, indicating a rejection of niche equivalency) and typically occupy different habitat niches (*p* = 0.113, indicating a rejection of niche similarity) (Figure 5).

## 4 Discussion

The coexistence of closely related species in overlapping ranges creates a fascinating ecological dynamic, especially in light of anthropogenic alterations to their habitats. The anthropogenic fragmentation of grassland ecosystems in Inner Mongolia, China, offers a unique opportunity to study how Jankowski's Bunting and Meadow Bunting adapt to changing habitats due to land‐use conversion and agricultural intensification. This study focuses on Jankowski's Bunting and Meadow Bunting, whose geographical distributions largely overlap within the steppe ecosystems of Inner Mongolia. Previous studies primarily described the population sizes and nesting ecologies of these species (Deng et al. 2003; Choi et al. 2020; Zhang et al. 2022). We quantitatively assessed their breeding habitat selection and niche overlap to gain insights into the ecological requirements of each species and their potential for competition or coexistence in a shared environment (Kosiński and Tryjanowski 2000).

Consistent with previous research on habitat selection in steppe birds, our results indicate that vegetation structure and habitat configuration are crucial determinants of breeding habitat selection for these two buntings (Benítez‐López et al. 2017; Gómez‐Catasús et al. 2019). The two sister species exhibited distinct habitat preferences. Jankowski's Bunting preferred areas with higher plant cover and height, as well as greater core area and largest patch index. In contrast, Meadow Bunting tended to inhabit sites with lower vegetation density but higher edge and patch density. These differences in habitat preferences are likely driven by species‐specific ecological requirements and adaptations. As an endangered species with a limited breeding range, Jankowski's Bunting may be more sensitive to habitat degradation and fragmentation (Han et al. 2020). Consequently, it selects areas with higher vegetation cover and structural complexity that provide better nesting and foraging opportunities (Zhang et al. 2022). In contrast, Meadow Bunting, which has a wider distribution range and a stable population trend, may exhibit greater habitat plasticity and tolerance to disturbances. This adaptability enables it to utilize a broader range of habitats, including more open and disturbed areas (BirdLife International 2024a). Additionally, landscape‐scale variables influenced habitat selection, emphasizing the importance of considering fine‐scale habitat configuration and fragmentation in conservation planning for these species.

The observed non‐linear responses of both buntings to habitat variables highlight the complexity of species‐habitat relationships. Our GLM analysis revealed that both Jankowski's Bunting and Meadow Bunting exhibited unimodal responses to habitat gradients, indicating optimal habitat conditions beyond which occurrence probabilities decline. These non‐linear patterns suggest that habitat suitability for these species depends on specific thresholds or ranges of habitat variables, rather than following simple linear relationships (Jamil and Ter Braak 2013; Anderson et al. 2022). Identifying these thresholds is essential for effective habitat management and conservation planning, as it enables the delineation of critical habitat areas and the establishment of conservation priorities (Salgueiro et al. 2018). For instance, maintaining habitat conditions within the optimal range identified for Jankowski's Bunting—such as areas with moderate vegetation cover and structural complexity—could enhance breeding success and population persistence. Similarly, identifying areas where Meadow Bunting occurrence peaks can guide habitat restoration efforts and inform land‐use planning to minimize adverse impacts on this species. Furthermore, the presence of concave‐downward response patterns in the response surfaces suggests that changes in habitat quality may have diminishing effects on species occurrence, highlighting the potential resilience of these species to moderate habitat degradation (Donaldson et al. 2019; McCloy et al. 2022). Unrecorded variables, such as soil type and microhabitats, may account for further variation in species occurrence probability. For example, heavy clay soils, which tend to retain more moisture than free‐draining soils, may be less suitable for species that rely on drier conditions for foraging or nesting (Kosiński and Tryjanowski 2000). T This difference in soil moisture levels can influence vegetation composition and structure, subsequently affecting breeding habitat suitability for various species.

The niche overlap analysis provides insights into the ecological coexistence of two closely related bird species within the steppe ecosystems of Inner Mongolia. The endangered Jankowski's Bunting exhibits a narrower niche range and higher specialization, indicating a greater dependency on specific habitat characteristics for survival and reproduction. In contrast, the Meadow Bunting, as a generalist species, displays a broader niche range along the two PCA axes, suggesting more flexible habitat and resource use. Despite occupying similar geographical ranges and exhibiting some degree of niche overlap, these species seldom share identical niches (Figure 5; Figure S2), indicating niche differentiation that minimizes competition for resources. Significant differences in niche centroids further underscore the distinct habitat preferences of these buntings, suggesting that they may partition resources along environmental gradients to reduce interspecific competition (Ayebare et al. 2023). This aligns with studies showing that temporal and spatial variability in niche differences among closely related taxa can promote species co‐occurrence (García‐Navas et al. 2021; Chiatante 2022; Petalas et al. 2024). For instance, a study focusing on five sympatric bunting species revealed high niche overlap in breeding habitat use; however, each species exhibited differences in preferred habitats based on various parameters, functionally segregating habitats and contributing to species co‐occurrence (Heim et al. 2022). Nevertheless, the observed niche overlap implies that competition for shared resources may still occur, particularly in areas where suitable habitats are limited. Therefore, conservation efforts should focus on preserving habitat diversity and integrity to accommodate the varying needs of coexisting species and mitigate potential competitive interactions.

## 5 Implications for Conservation

The conservation implications of our findings extend beyond the two species studied to broader management strategies, particularly in light of ongoing habitat degradation and anthropogenic disturbances in steppe ecosystems. Steppe environments are characterized by vast expanses of grassland and relatively low rainfall, making resources such as food, water, and nesting sites critical for the survival and reproduction of their inhabitants. In these habitats, birds that use different resources or occupy distinct niches can coexist more readily than those with overlapping ecological requirements (Traba et al. 2015). The observed differences in habitat preferences and niche differentiation suggest that conservation strategies should account for species‐specific requirements and adaptability to habitat changes. The distribution of Jankowski's Bunting has markedly contracted due to habitat loss and degradation in recent decades, while a century‐long decline in the breeding population of Meadow Bunting has been observed in South Korea (Han et al. 2018; Choi et al. 2020). Given rising conservation concerns, preserving and restoring habitats with diverse vegetation structures and maintaining landscape connectivity are crucial for safeguarding the breeding habitats of both Jankowski's Bunting and Meadow Bunting. Conservation efforts should prioritize the protection of intact grasslands and shrublands, as well as the restoration of degraded habitats through targeted management practices, including rotational grazing and habitat restoration initiatives. Furthermore, landscape‐level conservation planning should aim to minimize habitat fragmentation and enhance habitat connectivity to facilitate species dispersal and gene flow, particularly for the endangered Jankowski's Bunting. Further research incorporating long‐term monitoring is necessary to evaluate the effectiveness of conservation interventions and track population trends of these vulnerable bird species in Inner Mongolia's grassland ecosystems.

## Author Contributions

**Zheng Han:** conceptualization (equal), data curation (equal), formal analysis (equal), investigation (equal), writing – original draft (equal), writing – review and editing (equal). **Xi Yang:** formal analysis (equal), investigation (equal), visualization (equal), writing – original draft (equal). **Lishi Zhang:** formal analysis (equal), investigation (equal), visualization (equal), writing – original draft (equal). **Frédéric Jiguet:** conceptualization (equal), formal analysis (equal), methodology (equal), writing – original draft (equal), writing – review and editing (equal). **Piotr Tryjanowski:** conceptualization (equal), methodology (equal), writing – original draft (equal), writing – review and editing (equal). **Haitao Wang:** conceptualization (equal), formal analysis (equal), methodology (equal), writing – original draft (equal), writing – review and editing (equal).

## Conflicts of Interest

The authors declare no conflicts of interest.

## Supporting information


Data S1.

## Data Availability

Data could be found at https://github.com/Shenggeqianxing/niche‐overlap.
