# Supplementary material for: Niche Overlap Between Two Sympatric Steppe Birds in Inner Mongolia: Habitat Selection and Insights for Conservation
Source: Ecol Evol. 2025 Feb 24;15(2):e71010. doi: 10.1002/ece3.71010 (PMC11850443; doi:10.1002/ece3.71010)
Supplement: Supplementary file 1 — Data S1. [file ECE3-15-e71010-s001.docx]

**Table S1.** Mean values and Standard Deviation of breeding habitat variables inhabited by Jankowski’s Bunting and Meadow Bunting. With ANOVA test, variables that significantly differed between the two groups (p<0.01) are shown in boldface.

|  | Jankowski’s Bunting  (N=70) | | Meadow Bunting  (N=58) | | ANOVA  Test |
| --- | --- | --- | --- | --- | --- |
|  | Mean | SD | Mean | SD | p-value |
| Plant richness | 5.03 | 1.82 | 5.26 | 1.85 | 0.48 |
| Plant cover | 0.73 | 0.15 | 0.67 | 0.24 | 0.075 |
| **Plant height** | 20.04 | 10.39 | 12.38 | 7.97 | **<0.001** |
| Grassland proportion | 0.72 | 0.34 | 0.73 | 0.25 | 0.75 |
| **Edge density** | 30.57 | 30.69 | 55.46 | 43.84 | **<0.001** |
| **largest patch index** | 87.32 | 16.56 | 77.7 | 19.7 | **0.003** |
| **Patch density** | 11.43 | 9.14 | 16.91 | 12.03 | **0.004** |
| **Shannon diversity** | 0.28 | 0.28 | 0.47 | 0.34 | **<0.001** |
| **Total core area** | 22.65 | 3.17 | 19.99 | 4.48 | **<0.001** |
| **NDVI** | 102.16 | 10.53 | 115.43 | 13.55 | **<0.001** |

**Table S2** The explanation of landscape variables assessed at each sampling site in Inner Mongolia.

| **Variable** | **Explanation** |
| --- | --- |
| Edge  density | Measures the total length of the boundaries between different patch types within a landscape, normalized by the total landscape area. Higher edge density indicates a more fragmented landscape. More edges mean more transitions between different land-cover types, suggesting fragmentation. |
| Patch  density | The number of patches of a particular land-cover type per unit area of the landscape. Higher patch density reflects a more fragmented landscape, where the landscape is divided into smaller, isolated patches of a given land-cover type. |
| Shannon  diversity | A metric that quantifies the diversity of land-cover types within a landscape. It combines both the number of land-cover types (richness) and their relative abundance (evenness) into a single value. H′=−∑(p_i​_ lnp_i_​), where p_i_​ is the proportion of each land-cover type. Higher values indicate greater diversity in the landscape, while lower values indicate more homogeneity. |
| grassland proportion | The proportion of grassland within a 300-meter radius of each sampling point. |
| Total  Core  area | The total area of habitat core within a landscape. Habitat core is the interior portion of a patch, typically defined as being at least a certain distance (e.g., 100 meters) from the edge. Larger core areas are often considered high-quality habitat because they are less influenced by edge effects and more suitable for many species. |
| Largest  patch  index | The proportion of the landscape occupied by the largest patch of a particular land-cover type. A higher value suggests that the largest patch dominates the landscape, which may indicate less fragmentation or the dominance of one land-cover type in the landscape. |

**Table S3** Principal Component Analysis (PCA) results based on 10 habitat variables assessed at each bird sampling point in Inner Mongolia. The explained variance, eigenvalue, and factor loadings of the first five principal components (i.e. Dim.1- Dim.5) are shown.

|  | Dim.1 | Dim.2 | Dim.3 | Dim.4 | Dim.5 |
| --- | --- | --- | --- | --- | --- |
| eigenvalue | 4.43 | 1.73 | 1.02 | 0.98 | 0.68 |
| variance_percent | 44.26 | 17.26 | 10.16 | 9.77 | 6.76 |
| plant_richness | -0.11 | -0.73 | 0.00 | 0.04 | -0.63 |
| plant_cover | -0.10 | -0.75 | 0.01 | 0.05 | 0.50 |
| plant_height | -0.11 | -0.65 | -0.46 | 0.28 | 0.11 |
| grassland_proportion | -0.05 | -0.24 | 0.87 | 0.35 | 0.04 |
| edge_density | 0.98 | -0.07 | 0.00 | 0.02 | -0.03 |
| largest patch index | -0.90 | 0.05 | -0.01 | 0.11 | -0.06 |
| patch_density | 0.87 | -0.05 | -0.02 | 0.12 | -0.07 |
| shannon_diviversity | 0.95 | -0.04 | 0.02 | -0.04 | 0.03 |
| total_core_area | -0.98 | 0.07 | 0.00 | 0.00 | 0.01 |
| NDVI | -0.07 | -0.39 | 0.20 | -0.87 | 0.03 |


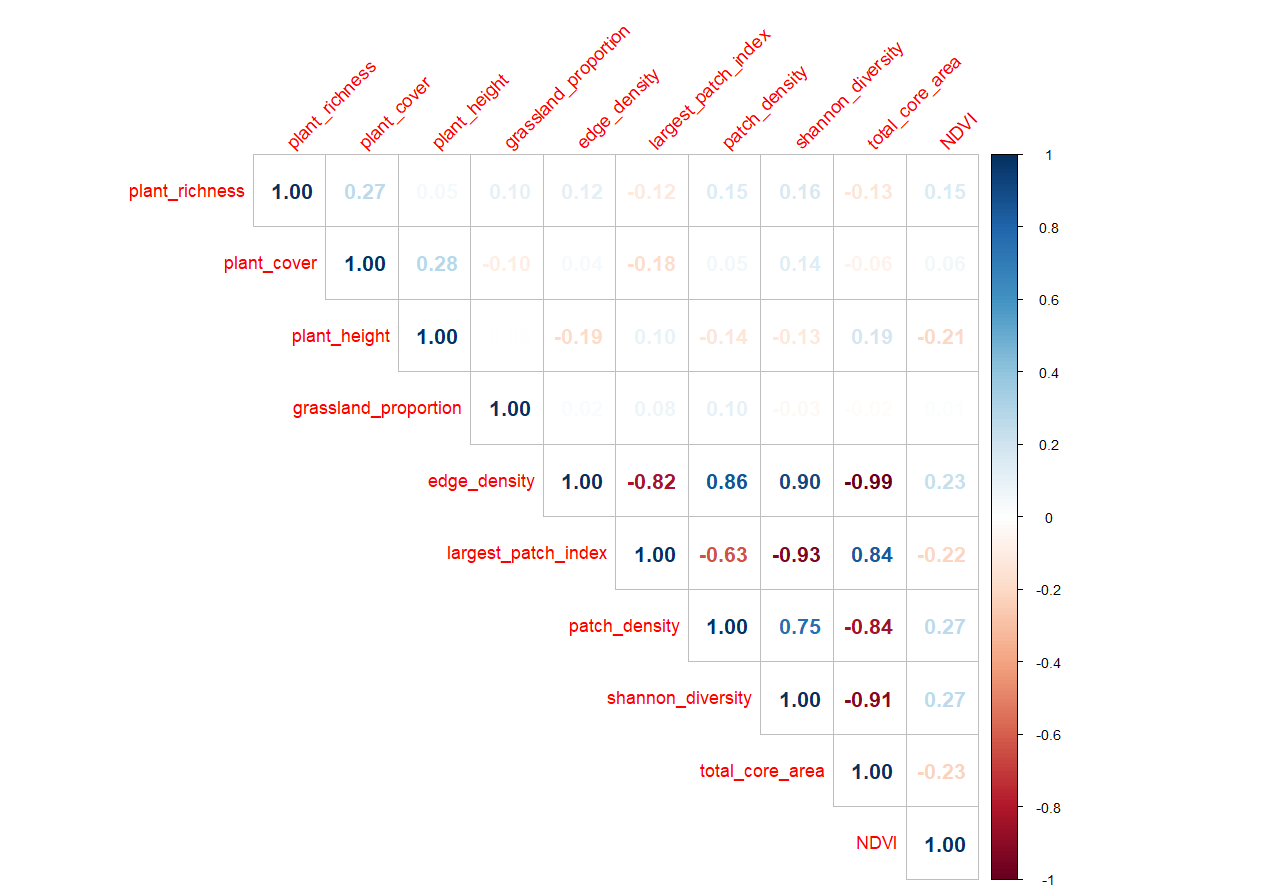


**Figure S1.** Heatmap to show the correlation matrix among the ten habitat variables. Positive correlations are displayed in blue and negative correlations in red color. The values of Pearson correlation coefficients are shown in text.


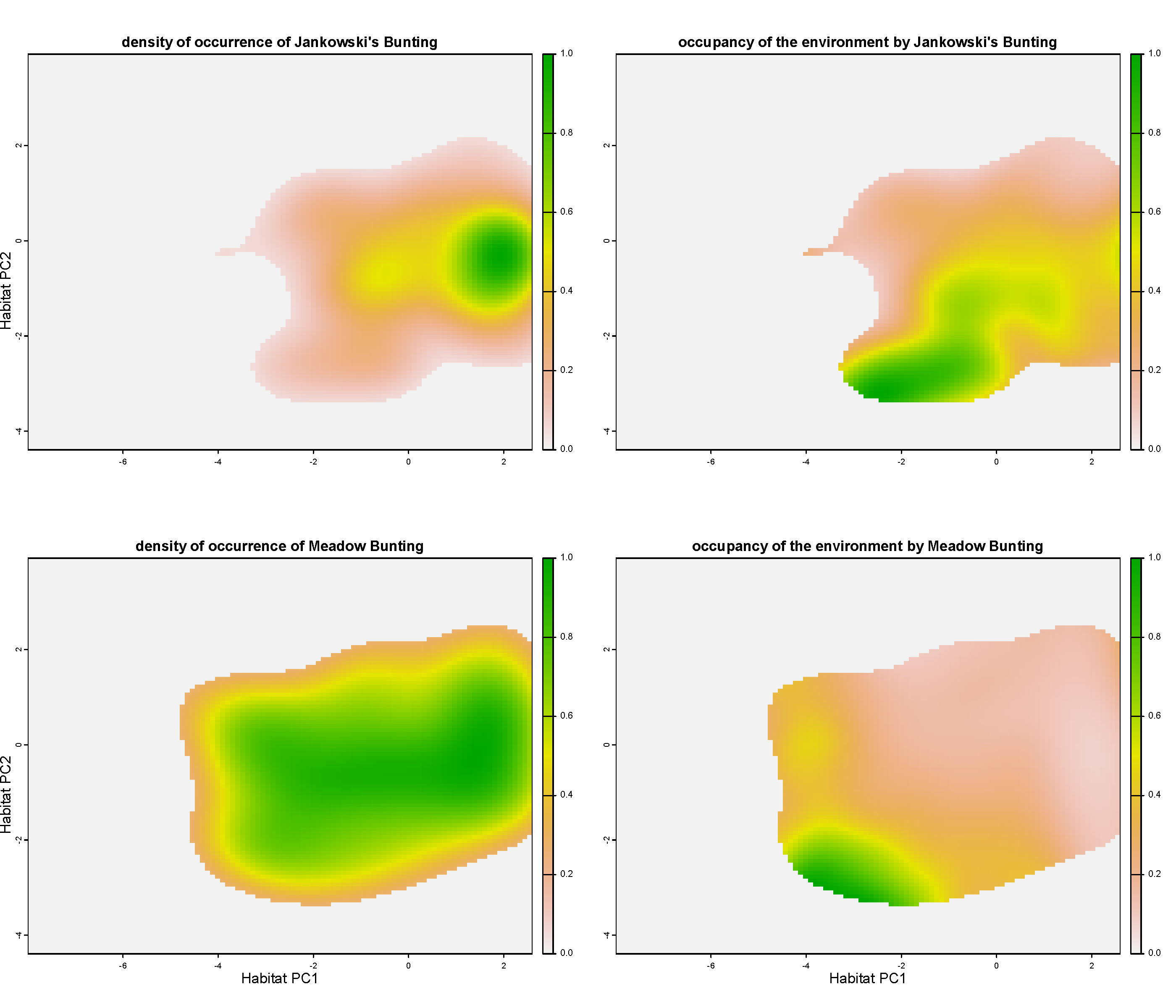


**Figure S2** The occurrence density of Jankowski’s Bunting and Meadow Bunting along the two first axes of the habitat PCA and the occupancy of the available environment by each species (density of occurrences divided by the density of environment in the study area).
